# Case Report: Bupropion Reduces the [^123^I]FP-CIT Binding to Striatal Dopamine Transporter

**DOI:** 10.3389/fpsyt.2021.631357

**Published:** 2021-02-22

**Authors:** Ivan Milenkovic, Lucie Bartova, Konstantinos Papageorgiou, Siegfried Kasper, Tatjana Traub-Weidinger, Dietmar Winkler

**Affiliations:** ^1^Department of Neurology, Medical University of Vienna, Vienna, Austria; ^2^Department of Psychiatry and Psychotherapy, Medical University of Vienna, Vienna, Austria; ^3^Division of Nuclear Medicine, Department of Biomedical Imaging and Image-guided Therapy, Medical University of Vienna, Vienna, Austria

**Keywords:** depression, parkinsonism, bupropion, FP-CIT, dopamine transporter

## Abstract

The diagnosis of parkinsonian syndromes in patients with severe depression may be challenging due to overlapping clinical phenomena, especially regarding psychomotor and affective symptoms. [^123^I]FP-CIT-SPECT is a useful method to detect degenerative parkinsonian disorders. However, some drugs may influence the tracer binding and thus alter the result. We present a case of 56-year-old female inpatient with difficult-to-treat late-onset depression. Since the current major depressive episode (MDE) was accompanied by psychotic features including delusions and hallucinations as well as hypokinesia, stooped posture and hypomimia, underlying degenerative parkinsonism was suspected. The pathologic [^123^I]FP-CIT-SPECT scan under ongoing antidepressant therapy with bupropion 300 mg/die (serum level of bupropion 43 ng/ml and hydroxybupropion 2,332 ng/ml) showed reduced [^123^I]FP-CIT binding throughout the striatum. The scan normalized upon a wash-out phase of four half-time periods (serum level of bupropion was 0.4 ng/ml and for hydroxybupropion 80.5 ng/ml). Our report should serve as a cautionary note for use of [^123^I]FP-CIT in depressed patients, particularly in those treated with drugs interfering with the dopamine transporter. Furthermore, our case argues for a need of consultation of a movement disorder specialist prior to dopamine transporter imaging.

## Introduction

The diagnosis of parkinsonian syndromes in patients suffering from severe depression can be challenging. On one hand, these patients may exhibit hypomimia-like facial expression, parkinsonian-like posture or develop psychotic features (1). On the other hand, depression as the most common non-motor symptom in Parkinson's disease (PD) precedes the motor symptoms for ~10 years (2). In doubtful cases, [^123^I]FP-CIT-SPECT is a useful tool to detect decrease in striatal binding to dopamine transporters (DAT) (3) due to loss of nigrostriatal terminals (4). However, commonly prescribed psychopharmacotherapeutics that bind to DAT may change the binding properties of the tracer (5) and hence, alter the results (6). Although several drugs have been identified, occasionally patients and physicians are not aware that these drugs ought to be paused prior to [^123^I]FP-CIT-SPECT imaging. This results either in prolonging the diagnostic procedures or, if not considered by specialists, even in imprecise diagnosis. Here we report on a prominent effect of short-term bupropion therapy on [^123^I]FP-CIT-SPECT imaging.

## Case Presentation

The 56-year-old female inpatient (weight: 70 kg, height: 160 cm) had almost a 1-year history of a difficult-to-treat depression (7) with late onset (8). In the course of the current severe major depressive episode (MDE) with psychotic symptoms (International Classification of Diseases, 10th revision, ICD-10 (9): F32.3) that represented her first MDE so far, the patient was anhedonic and exhibited indecisiveness, feelings of hopelessness and worthlessness as well as excessive guilt and diminished ability to concentrate. In the course of her subjective cognitive decline, the patient exhibited a total score of 28/30 at the Mini-Mental State Examination (MMSE) (10) and of 15/18 at the DemTect (11) representing values within the normal range. Furthermore, she suffered from psychomotor slowing, loss of interest and pleasure affecting her hobbies and nearly all activities including the necessary daily routine, as well as delusions and hallucinations. She negated suicidal ideation and there was no suicidal history. The patient showed a total score of 46 at the Montgomery-Åsberg Depression Rating Scale (MADRS) (12) and 6 at the Clinical Global Impressions Scale (CGI-S) (13). In terms of psychiatric and somatic comorbidities, the patient suffered from nicotine dependence (ICD-10: F17.2) and psoriasis vulgaris (ICD-10: L40.0).

The antidepressant combination treatment with mirtazapine 45 mg and venlafaxine 375 mg was expanded with vortioxetine 10 mg/die, but failed to improve the symptoms. Neither augmentation with second-generation antipsychotics (aripiprazole 20 mg/die) (14) and S-ketamine (15) that was administered 2–3 times/week intravenously (up to 50 mg per infusion), nor a series of 11 electroconvulsive therapies (ECT) with subsequent regular maintenance ECTs, all with bilateral stimulation up to 100%, improved the severe depressive symptoms (16).

**Figure 1:**
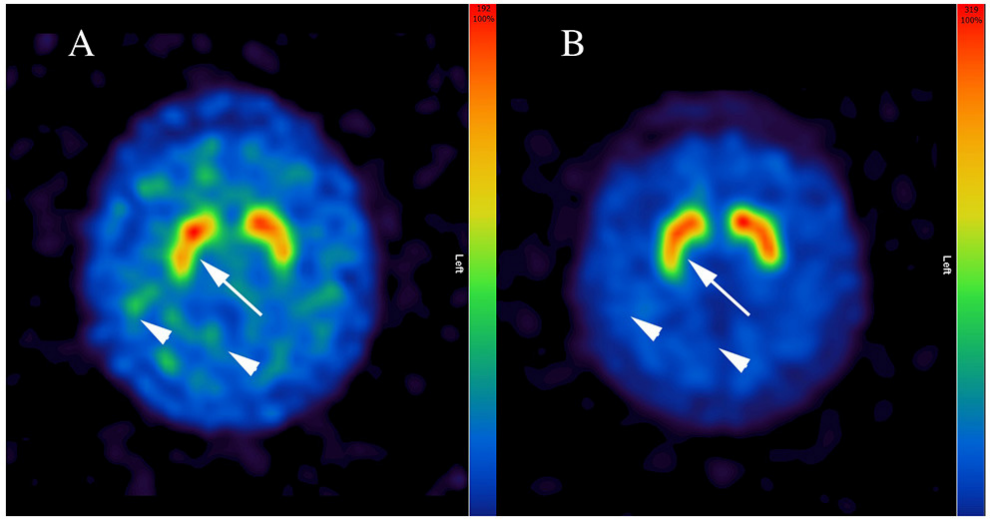
Representative [^123^I]FP-CIT-SPECT images of the patient. Note inhomogeneous and reduced dopamine transporter binding of the radiopharmaceutical in putamen bilaterally (arrow in **A**) and low contrast between striatal and cortical binding during short-term bupropion therapy (arrowheads in **A**), which normalized after a wash-out phase of four half-life periods (arrow and arrowheads in **B**). Bupropion reduced the binding of [^123^I]FP-CIT up to 45% (i.e., in right putamen).

Due to massive anhedonia, progressive hypomimia, general slowness of movements, delusions and hallucinations, a suspicion of degenerative parkinsonism was raised. Since there were no postural instability and no history of falls, dementia with Lewy bodies (DLB) was considered as a differential diagnosis. While the cranial magnetic resonance imaging was unremarkable, [^123^I]FP-CIT-SPECT was initiated. Four days prior to the [^123^I]FP-CIT-SPECT examination the antidepressant treatment was adapted adding bupropion 150 mg/die that was increased to 300 mg/die on the day of [^123^I]FP-CIT-SPECT. An intravenous bolus injection of ~185 MBq [^123^I]FP-CIT according to GMP criteria was applied ~1 h after the medication intake and ~3 h before the acquisition of the scans. A three-headed Gamma camera (IRIX 465, Philips Medical System) system was used for SPECT Imaging (3° steps/360°, 60 s per step, 40 frames, pixel size of 3,50 mm, pixel matrix of 128 × 128, LEHR-PAR collimator). OSEM reconstruction (5 subsets, 16 iterations), post-filtering with 3 D Gauss filter (FWHM 7 cm) and Chang's attenuation correction (17) with an attenuation coefficient of 0.15 for all images were performed. [^123^I]FP-CIT-SPECT was reported pathologic with reductions of striatal DAT binding bilaterally (arrow in Figure 1A) and low contrast between striatal and cortical binding (arrowheads in Figure 1A). The specific binding ratio was calculated using occipital region as a reference (18), and the standard deviations from the reference value mean (Z-score) were calculated using BRASS HERMES software containing age-corrected control population: left caudate nucleus 1.77 (Z-Score −4.2), right caudate 1.81 (Z-Score −3.91), left putamen 1.68 (Z-Score −3.65), and right putamen 1.54 (Z-Score −4.08).

Subsequently, consultant neurologist was involved revealing general slowness of movements without true bradykinesia with bilateral reduction of arm pendular movement upon walking. Rigidity was not detected, but active resistance. Importantly, the ongoing therapy with bupropion was noted, whereby the serum blood levels of bupropion (43 ng/ml) and the active metabolite hydroxybupropion (2,332 ng/ml) measured on the day of the neurologic examination were within the therapeutic range. Once the repetition of [^123^I]FP-CIT SPECT after a wash-out phase was suggested, bupropion was discontinued for 8 days (half-life period 20–37 h). At the day of [^123^I]FP-CIT-SPECT rescan, the serum blood level for bupropion was 0.4 ng/ml and for hydroxybupropion 80.5 ng/ml. [^123^I]FP-CIT SPECT was reported normal (Figure 1B) using the same analysis, with overall normalization of specific binding ratios and Z-scores: left caudate nucleus 3.18 (Z-score −0.2), right caudate nucleus 3.28 (Z-score −0.63), left putamen 3.08 (Z-score −0.51) and right putamen 2.94 (Z-score −0.05).

In the course of the comprehensive differential diagnostics, laboratory tests including blood, urine and cerebrospinal fluid, electroencephalography, and [^18^F]-FDG PET/CT were performed revealing largely unremarkable results. After degenerative parkinsonism, dementia, autoimmune encephalitis, and other degenerative and inflammatory processes could be excluded, further therapeutic optimization was undertaken. While the antidepressant treatment with venlafaxine and vortioxetine was discontinued, tranylcypromine was initiated after the necessary wash-out phase. While staying on a low-tyramine diet, tranylcypromine was well-tolerated by the patient who partially responded to this treatment adaptation. Subsequently, tranylcypromine was slowly increased up to 50 mg/die. Moreover, the patient received clozapine as additional augmentation treatment that was gradually optimized up to 100 mg/die. Under a treatment regimen including tranylcypromine 50 mg, mirtazapine 30 mg, clozapine 100 mg, aripiprazole 10 mg, and clonazepam 2 mg/die that was accompanied by regular psychotherapeutic-, physiotherapeutic and ergotherapeutic support, a remarkable reduction of her depressive and psychotic symptoms was achieved. The adherent patient showed a total score of 28 at the MADRS and of four at the CGI-S and could be discharged from psychiatric inpatient care. Continuing the abovementioned psychopharmacotherapy she was subsequently treated as outpatient and received psychosocial support at home.

## Discussion

The present case report could show effect of blood serum levels of bupropion and its active metabolite hydroxybupropion on [^123^I]FP-CIT binding to DAT. Hereby, therapeutic doses of bupropion reduced the binding of [^123^I]FP-CIT ~45–50% throughout the striatum, that was reversible after four half-life periods of bupropion. Since there was no further change in the ongoing therapy, in particular concerning venlafaxine and vortioxetine or mirtazapine, we attribute the observed effect to bupropion. Bupropion is a dose-dependent norepinephrine-dopamine reuptake inhibitor (19), with high occupancy at higher doses. Previous studies suggested changed DAT binding properties in depressed individuals. However, it is still a matter of debate, whether the primary mechanism is down-regulation (19) or up-regulation of DAT (20). Thus, concomitant therapy with bupropion may lead to a significant loss of [^123^I]FP-CIT binding and hence, might result in false positive diagnosis of degenerative parkinsonism. The [^123^I]FP-CIT binding pattern in our patient, however, appeared atypical for idiopathic PD and DLB (21), showing a reduced DAT binding throughout the striatum. Indeed, a recent paper noted similar observation, although some asymmetry in [^123^I]FP-CIT binding in a patient under bupropion therapy was reported (22). Thus, careful visual examination of DAT scan in a patient under dopaminergic medications, such as bupropion, may be critical (5). Taken together, our data suggest, that a discontinuation of bupropion of at least four half-life periods is necessary prior to [^123^I]FP-CIT scan. However, further prospective studies are needed to examine whether longer discontinuation is necessary, as suggested by some authors (22). Our report should serve as a cautionary note for use of [^123^I]FP-CIT in depressed patients, particularly in those treated with drugs interfering with DAT. Furthermore, our case argues for a need of consultation of a movement disorder specialist prior to DAT imaging. This might be of crucial importance especially in patients suffering from late onset depression that was recently related to elevated rates of motor and non-motor symptoms known from degenerative parkinsonian syndromes as well as to altered [^123^I]FP-CIT binding and, hence, to increased risk of PD and DLB (8).

## Data Availability Statement

The original contributions presented in the study are included in the article/supplementary material, further inquiries can be directed to the corresponding author.

## Ethics Statement

Written informed consent was obtained from the individual(s) for the publication of any potentially identifiable images or data included in this article.

## Author Contributions

IM and LB were responsible for clinical care and data analysis and drafted the manuscript. KP, DW, and SK supervised clinical care and critically revised the manuscript. TT-W critically revised the manuscript and performed and analyzed [^123^I]FP-CIT-SPECT scans. All authors contributed to the article and approved the submitted version.

## Conflict of Interest

IM received grant from Takeda. LB received travel grants and consultant/speaker honoraria from AOP Orphan, Medizin Medien Austria, Vertretungsnetz, Schwabe Austria, Janssen, and Angelini. KP received honoraria from AOP Orphan Pharmaceuticals, Germania Pharmaceuticals, Lundbeck, and Janssen-Cilag. SK received grants/research support, consulting fees, and/or honoraria from Angelini, AOP Orphan Pharmaceuticals, Celegne GmbH, Eli Lilly, Janssen-Cilag Pharma GmbH, KRKA-Pharma, Lundbeck A/S, Mundipharma, Neuraxpharm, Pfizer, Sanofi, Schwabe, Servier, Shire, Sumitomo Dainippon Pharma Co. Ltd. and Takeda. DW received lecture fees/authorship honoraria from Angelini, Lundbeck, and Medizin Medien Austria. The remaining author declares that the research was conducted in the absence of any commercial or financial relationships that could be construed as a potential conflict of interest.
